# Bitcoin, Libra und digitale Zentralbankwährungen — ein Geldsystem der Zukunft?

**DOI:** 10.1007/s10273-020-2743-y

**Published:** 2020-09-25

**Authors:** Jonas Groß, Bernhard Herz, Jonathan Schiller

**Affiliations:** Rechts- und Wirtschaftswissens. Fakultät, Universität Bayreuth, Universitätsstraße 30, 95447 Bayreuth, Deutschland

**Keywords:** E42, E52, G28

## Abstract

Seit der Einführung von Bitcoin 2008 und vieler anderer Kryptowährungen ist eine neue Welle innovativer Zahlungsprojekte im Gange, darunter Innovationen wie Libra — als supranationaler Stablecoin konzipiert — und digitale Zentralbankwährungen. Diese privaten und öffentlichen Projekte sind durch verschiedene Wechselbeziehungen miteinander verbunden. Bitcoin hat sich bislang nicht zu einem weit verbreiteten Zahlungsmittel entwickelt, unter anderem wegen der erheblichen Preisvolatilität. Im Gegensatz dazu soll Libra ein eher konventionelles Zahlungsmittel mit engen Beziehungen zum bestehenden Bankensektor werden, was viele Fragen zur Geld- und Finanzstabilität aufwirft. Digitale Zentralbankwährungen könnten als Reaktion des öffentlichen Sektors auf diese privaten Projekte angesehen werden, um die vorherrschende Rolle der Zentralbanken im Währungssystem der Zukunft zu sichern.

## Abstract (en)

After the launch of Bitcoin in 2008 and the subsequent introduction of more than 6,600 cryptocurrencies, a new wave of innovative payment projects is currently on its way, including innovations like Libra — designed as a supranational stable coin — and central bank digital currencies (CBDCs). Various interrelations link these private and public projects. Contrary to the original intentions, Bitcoin has not developed into a widespread means of payments, not the least due to its considerable price volatility. Its most significant contribution could be the „proof of concept“ for an innovative, private means of payment outside the conventional monetary system. In contrast, Libra is designed as a rather conventional means of payment with close relations with the existing banking sector, which raises numerous policy questions concerning monetary and financial stability. Central bank digital currencies could be viewed as a public sector response to these private projects to secure central banks’ predominant role in the monetary system of the future.

Seit der Einführung von Bitcoin im Jahr 2008 hat sich die Digitalisierung des Geldes dynamisch weiterentwickelt. Die revolutionäre Technologie hinter Bitcoin, die sogenannte Distributed-Ledger-Technologie (DLT), ermöglicht es, Geld- und Zahlungssysteme neu zu denken. Inzwischen gibt es nicht nur über 6.600 Kryptowährungen, sondern weitere darauf aufbauende Projekte, die von Öffentlichkeit, Zentralbanken und Regulatoren aufmerksam verfolgt werden. Dies gilt insbesondere auch für die neue supranationale Währung Libra, die von einem Konsortium rund um Facebook im Jahr 2019 angekündigt wurde. Sie soll wertstabil sein, grenzüberschreitende Zahlungen verbilligen und die finanzielle Inklusion erhöhen. Auch der öffentliche Sektor hat das Potenzial der neuen Technologien und die Notwendigkeit der weiteren Digitalisierung des Geldsystems erkannt. So forschen weltweit Zentralbanken wie die Europäische Zentralbank (EZB) und die US-amerikanische Federal Reserve über Möglichkeiten und Risiken von digitalem Zentralbankgeld (Central Bank Digital Currency, CBDC). Die chinesische Notenbank hat im April 2020 bereits die Pilotphase ihres CBDC-Projekts DC/EP gestartet und die Testläufe zuletzt noch deutlich ausgeweitet.

**Jonas Groß**, M. Sc., ist wissenschaftlicher Mitarbeiter an der Universität Bayreuth und am Frankfurt School Blockchain Center.

**Prof. Dr. Bernhard Herz** ist Lehrstuhlinhaber für Geld und Internationale Wirtschaft an der Universität Bayreuth.

**Jonathan Schiller**, M. Sc., ist wissenschaftlicher Mitarbeiter an der Universität Bayreuth.

Zwischen diesen Projekten des privaten und öffentlichen Sektors bestehen vielfältige Wechselbeziehungen. Private Unternehmen bieten auf Basis der neuen Technologien innovative Zahlungsformen an. Notenbanken und Regierungen werden dadurch angetrieben, selbst neue Zahlungsmittel zu entwickeln, bevor private Alternativen entstehen, auf die sie nur begrenzten Einfluss ausüben können und welche die Finanzmarktstabilität gefährden könnten. Aufgrund von First-Mover-Vorteilen entwickelt sich dieses Wechselspiel außerordentlich dynamisch.

Damit stellt sich eine Reihe grundsätzlicher Fragen für die zukünftige Geldordnung: Wird es beim Nebeneinander von staatlichem und privatem Geld bleiben? Wie können Zentralbanken und Regulierungsbehörden monetäre Stabilität sicherstellen, wenn Transaktionen zunehmend in privaten Geldsystemen abgewickelt werden sollten? Verlieren derzeit dominierende Fiat-Währungen, wie der US-Dollar, durch die neuen Geldformen international an Bedeutung?

Aus ordnungspolitischer Perspektive ist Geld zunächst ein Produkt wie jedes andere auch. Kosten und Nutzen bestimmen Angebot und Nachfrage; technologische Neuerungen sind wichtige Treiber für Produkt- und Prozessinnovationen. Im Zuge der zunehmenden Arbeitsteilung, Spezialisierung und Digitalisierung entstehen neue Formen des Geldes wie Bitcoin und neue Zahlungsdienstleister wie PayPal. Gleichzeitig ist Geld aufgrund seiner zentralen Stellung im Wirtschaftsprozess aber auch ein besonderes Produkt, dessen Herstellung und Verwendung durch Notenbanken und Regulierungsbehörden stark reguliert wird.

In diesem Zusammenspiel privater und öffentlicher Akteure sind verschiedene Geldformen entstanden. Zwei Merkmale sind dabei von besonderer Bedeutung (vgl. Tabelle 1). Zum einen können Zahlungsmittel danach abgegrenzt werden, ob Transaktionen wie im Falle von Bargeld Peer-to-Peer – also direkt zwischen den beiden beteiligten Parteien ohne Intermediäre – geleistet werden können oder ob eine zentrale Instanz für die Durchführung einer Zahlung benötigt wird. Zum anderen ist nach Art des Emittenten zwischen öffentlichem Zentralbankgeld und privat emittierten Geld zu unterscheiden. Geschäftsbanken emittieren komplementär zu den Zentralbanken private digitale Zahlungsmittel meist in Form von Bankdepositen mit dem Versprechen, diese jederzeit 1:1 in das gesetzliche Zahlungsmittel Bargeld umzutauschen.

Wie entwickelt sich das Geldsystem im Rahmen des aktuellen Innovationsschubs entlang verschiedener Dimensionen weiter? Wie differenziert es sich aus? Mit Bitcoin wurde erstmals ein dezentrales digitales Zahlungsmittel entwickelt, bei dem Transaktionen nicht durch einen einzigen bekannten und vertrauensvollen Intermediär, wie eine Bank, bestätigt werden, sondern von einem dezentralen Netzwerk von Computern. Zahlreiche weitere private Kryptowährungen sind dem Modell gefolgt und wurden wie Bitcoin basierend auf einer DLT kreiert. Inzwischen arbeiten auch viele Notenbanken an digitalem Zentralbankgeld, das für alltägliche Zahlungen verwendet werden kann, beispielsweise zwischen privaten Haushalten und Unternehmen. Im Bereich der Kryptowährungen sind vor allem private Nichtbanken als Anbieter aktiv. Ein neuer Ansatz ist das Libra-Projekt, das auf großes öffentliches Interesse gestoßen ist. Ein Konsortium privater Nichtbanken, darunter das Facebook-Tochterunternehmen Novi (ehemals Calibra) und andere technologieaffine Unternehmen wie Spotify und Uber, strebt hierbei an, ein privates digitales Zahlungsmittel zu entwickeln, das Transaktionen außerhalb des konventionellen Bankensystems ermöglicht. All diese Innovationen können weitreichende Folgen für das derzeitige Geldsystem und das Verhältnis zwischen privaten und öffentlichen Anbietern im Finanzsektor haben.

Tabelle 1
**Private und öffentliche Geldformen im Überblick**

| Emittent \ Ablauf der Transaktion | | Indirekt über Intermediär | Direkt Peer-to-Peer |
|---|---|---|---|
| Öffentlicher Sektor | Zentralbank | Zentralbank-reserven (CBDC) | Banknoten |
| Privater Sektor | Private Banken | Bankeinlagen | |
| | Private Nicht-Banken | Libra | Bitcoin |

Quelle: eigene Darstellung.

## Bitcoin adressiert Marktlücke

Mit Bitcoin wurde 2008 eine private Geldform geschaffen, die erstmals Zahlungen direkt (Peer-to-Peer), d. h. ohne Intermediäre wie Banken oder Zentralbanken, in digitaler Form ermöglicht. Im Bitcoin-System gibt es keine zentrale Instanz, die nach Durchführung einer Transaktion das entsprechende Konto der Transaktionspartner be- bzw. entlastet. Die eigentliche Innovation hinter Bitcoin steckt in der DLT – der Bitcoin-Blockchain – als technologischer Basis, die digitale Zahlungen ohne vertrauenswürdigen Intermediär erst möglich macht.

In der Bitcoin-Blockchain werden Transaktionen in Blöcken gesammelt und über kryptographische Verfahren mit der Transaktionshistorie verknüpft (Blockchain). Anders als im Falle einer Banküberweisung, die auf zentralisierten Datenbanken basiert, sind es im Bitcoin-System nicht zentrale Parteien, die Transaktionen auf Korrektheit überprüfen, sondern ein dezentrales Computernetzwerk bestehend aus zahlreichen sogenannten Minern. Konkret müssen Miner unter Aufwendung von Rechenleistung und Energie ein kryptographisches Rätsel lösen (Proof-of-Work). Wer diese Aufgabe als erstes erfüllt, erwirbt das Recht, die gesammelten Transaktionen zu bestätigen, und bekommt als Belohnung neben den Transaktionsgebühren eine bestimmte Zahl an Bitcoins ausgezahlt (Mining Reward). Aufgrund dieser dezentralen Konsensfindung ermöglicht das Bitcoin-System digitale Transaktionen ohne eine zentral-validierende Instanz.

Im Mai 2020 erreichte Bitcoin eine Marktkapitalisierung von ca. 185 Mrd. Euro (Coinmarketcap, 2020). Trotz der deutlich gestiegenen Verbreitung vor allem in Schwellen- und Entwicklungsländern sind in den letzten Jahren einige Konstruktionsprobleme des Bitcoin-Systems zu Tage getreten. Dies hat dazu geführt, dass Bitcoin in Industrieländern inzwischen eher weniger als globales Zahlungsmittel, sondern eher als digitaler Wertspeicher (digitales Gold) betrachtet wird. So ist das Bitcoin-Netzwerk in seiner jetzigen Form nur unzureichend skalierbar und kann nicht mehr als sieben Transaktionen pro Sekunde verarbeiten. Dies liegt daran, dass im Netzwerk im Durchschnitt nur alle 10 Minuten von den Minern ein Block hinzugefügt wird, sodass bei hohem Transaktionsaufkommen Zahlungen nur mit teils erheblichen Verzögerungen durchgeführt werden. Allerdings gibt es bereits Bestrebungen, die Transaktionsgeschwindigkeit und damit die Skalierbarkeit zu erhöhen, beispielsweise durch das sogenannte Lighting-Netzwerk.

Das Geldangebot im Bitcoin-System ist letztlich auf 21 Mio. Bitcoin-Einheiten begrenzt, wovon aktuell bereits ca. 18 Mio. im Umlauf sind (Coinmarketcap, 2020). Derzeit wächst die Bitcoin-Geldmenge um jährlich ca. 2 % und wird an die Miner in Form des Mining Rewards ausgezahlt. Dieser Mining Reward wird etwa alle vier Jahre halbiert, sodass sich die umlaufende Geldmenge bis zum Jahr 2140 asymptotisch der Höchstgrenze von 21 Mio. Bitcoin-Einheiten annähert. Aufgrund dieses starren Angebots werden Marktungleichgewichte allein über Änderungen der Nachfrage ausgeglichen, was mit einer erheblichen Preisvolatilität verbunden ist. Tägliche Kursanstiege bzw. -rückgänge von über 10 % sind keine Seltenheit.

## Was Libra von Bitcoin lernte

Inzwischen ist Bitcoin zwar in Ländern mit Weichwährungen, wie Argentinien oder Venezuela, ein relativ weit verbreitetes Zahlungsmittel, konnte sich aber aufgrund der oben beschriebenen Probleme, wie Skalierbarkeit, Volatilität und regulatorische Unsicherheit, nicht als globales Zahlungsmittel durchsetzen. Das im Sommer 2019 von Facebook initiierte Projekt „Libra“ möchte ein solches globales digitales Zahlungsmittel schaffen und die Probleme von Bitcoin überwinden. Libra basiert auch auf einer DLT, der sogenannten Libra-Blockchain. Sie wird von der Libra Association verwaltet, einer unabhängigen Organisation mit Sitz in der Schweiz. Im Unterschied zur Bitcoin-Blockchain soll die Technologie hinter Libra bis zu 1.000 Transaktionen pro Sekunde ermöglichen.

Libra ist als sogenannter Stablecoin konzipiert, also als wertstabile Kryptowährung. Die Wertstabilität soll dadurch erreicht werden, dass die Libra Association die durch den Verkauf von Libra erworbenen Mittel in Form von Bankeinlagen und kurzfristigen Staatsanleihen anlegt. Libra soll somit vollständig durch hochliquide, sichere Assets besichert werden – massive Wertschwankungen durch Veränderungen in Angebot und Nachfrage sind somit größtenteils ausgeschlossen. Gemäß des aktuellen Konzepts soll es im Libra-System zwei Geldformate geben. Zum einen sogenannte Single-Currency-Stablecoins, die durch eine Währung gedeckt sind, etwa Libra-Euro und Libra-Dollar, und zum anderen einen Multi-Currency-Stablecoin (Libra Coin), der durch einen Währungskorb gedeckt ist.

In Schwellen- und Entwicklungsländern mit tendenziell eher schwachen lokalen Währungen dürfte vor allem Libras Wertstabilität von Bedeutung sein. Hier könnte der Libra Coin als Währungskorb basierend auf starken Währungen wie dem Euro oder dem US-Dollar als ein attraktives Zahlungs- und Wertaufbewahrungsmittel dienen (Groß, Herz und Schiller, 2019). Allerdings ist in solchen Ländern bislang zu beobachten, dass als Parallelwährungen eher einzelne Währungen wie der US-Dollar und keine Währungskörbe verwendet werden.

In Industrieländern dürften vor allem die Single-Currency-Stablecoins von Interesse sein. Ein besonderer Vorteil der Libra ist dabei, dass sie auf einer DLT basiert und damit im Gegensatz zu konventionellen Währungen als „programmierbares“ Geld genutzt werden kann (Groß, Klein und Sandner, 2020). Somit könnten Libra-Coins programmiert werden, sodass bei Eintreten eines bestimmten Events X automatisch ein Folge-Event Y ausgelöst wird. Auf diese Art und Weise könnten beispielsweise Zinszahlungen oder sonstige Finanzflüsse automatisiert werden – Effizienzgewinne, die vor allem für die Industrie und Banken von Interesse sein dürften.

Zwar adressiert Libra eine Reihe von Problemen im Zusammenhang mit Bitcoin, offensichtlich gehen damit aber auch neue Besonderheiten einher, etwa der geringere Grad an Dezentralisierung und die (begrenzte) Kontrolle des Geldangebots. Das Geldangebot ist im Libra-System nicht wie im Falle von Bitcoin fixiert, sondern wird ausschließlich von der Nachfrageseite bestimmt: Steigt die Nachfrage nach Libra, wird im Zuge dessen auch das Libra-Geldangebot erhöht. Im Gegensatz zu Bitcoin, dessen langfristiger Wert durch eine strenge Kontrolle des Angebots gesichert werden soll, erfüllt diese Funktion bei Libra der fixe Wechselkurs zur Referenzwährung bzw. dem -währungskorb.

Das Libra-Netzwerk ist deutlich zentralisierter ausgelegt als das Bitcoin-Netzwerk. Bei Bitcoin entscheiden tausende Parteien über die Korrektheit von Transaktionen und die Governance des Systems. Bei Libra hingegen hat letztlich die Libra Association die Entscheidungsgewalt. Sie besteht derzeit aus 27 Partnern, wobei die Mitgliederzahl bis zur Einführung von Libra noch auf bis zu 100 ansteigen soll. Somit ist Libra zwar deutlich dezentraler ausgerichtet als Banken im klassischen Finanzsystem, allerdings weniger dezentral als Bitcoin.

Die Libra Association verfolgt für die Einführung von Libra einen straffen Zeitplan. Bei der Schweizerischen Finanzmarktaufsicht FINMA wurde bereits eine Lizenz zum Betreiben eines Zahlungssystems beantragt und Libra soll Ende 2020 eingeführt werden. Zwar wurden im Zuge der Überarbeitung des Konzepts (Libra 2.0) zahlreiche Kritikpunkte von Politik, Notenbanken und Bankenregulierung adressiert und betont, dass insbesondere die nationale Geldpolitik und die makroprudenzielle Politik nicht beeinträchtigt werden solle. Es wurde auch ein umfassendes Compliance-System angekündigt, um illegale Aktivitäten wie Geldwäsche und Terrorfinanzierung zu verhindern (Libra Association, 2020). Dennoch ist noch immer eine Reihe wichtiger Fragen zu klären. Offen sind insbesondere noch Fragen zur regulatorische Ausgestaltung, Datenschutz, Struktur der Transaktionsgebühren und Kapitalpuffer, um Krisensituationen abzufedern und die Finanzmarktstabilität zu sichern. Darüber hinaus ist bislang ungeklärt, wie schnell Transaktionen letztendlich abgewickelt werden und ob Zahlungen tatsächlich Peer-to-Peer durchgeführt werden können. Zwar wird die Libra Association aufgrund der Libra-Governance bei der Validierung der Transaktionen eine zentrale Rolle einnehmen. Allerdings sollen laut dem Konzeptpapier auch Peer-to-Peer-Zahlungen möglich sein (Libra Association, 2020).

## Reaktionen der Zentralbanken

Die rasanten Entwicklungen im Bereich von Kryptowährungen, Stablecoins und privaten digitalen Geldsystemen haben – nicht überraschend – die Zentralbanken auf den Plan gerufen. So formulierte Christine Lagarde in ihrer ersten Pressekonferenz als neue EZB-Präsidentin das Ziel, die Notenbank müsse „ahead of the curve" – diesen Innovationen also voraus – sein. Es gebe laut Lagarde eine Nachfrage insbesondere nach Stablecoins, die eine angemessene Reaktion der Zentralbank erfordere. Von zentraler Bedeutung für die EZB sind die Folgen der neuen Zahlungsformen und Geldsysteme für Finanzmarktstabilität und monetäre Analyse.

Im Fall von Bitcoin sind die Auswirkungen für die Zentralbanken und deren Politik überschaubar. Bitcoins werden außerhalb des konventionellen Finanzsektors kreiert und genutzt. Der sehr volatile Preis macht Bitcoin als Zahlungsmittel eher unattraktiv. Die Substitution konventioneller Zahlungsmittel durch Bitcoin ist daher nur in einem sehr begrenzten Umfang zu erwarten.

Inwiefern Libra für die Zentralbanken relevant werden könnte, ist derzeit noch offen und letztlich abhängig von der finalen Ausgestaltung. Der Multi-Currency-Libra-Stablecoin wird voraussichtlich ähnlich zu den Sonderziehungsrechten (SZR) des Internationalen Währungsfonds (IWF) als Währungskorb konzipiert. Die damit verbundenen Wechselkursrisiken und Wechsel- und Transaktionsgebühren machen eine Nutzung in Ländern mit stabilen Währungen relativ unattraktiv und würden wohl eine weite Verbreitung verhindern. Dabei ist allerdings zu beachten, dass Libra entsprechend der Reserve-Regelungen der Libra Association zu einem signifikanten Teil mit Euro-Bankdepositen und Euro-denominierten Staatsanleihen hinterlegt wäre. Das würde dazu führen, dass der Euro bei einer relativ weiten Verbreitung in Ländern mit schwächeren Währungen de facto weit über die Grenzen der Eurozone hinweg als Zahlungsmittel genutzt würde, und dass die Libra Association einen großen Teil der kurzfristigen Staatsanleihen stabiler Volkswirtschaften hielte. Dies birgt offensichtlich Risiken für die Finanzmarktstabilität.

Die Single-Currency-Libra-Stablecoins sollen in einem fixen Verhältnis in einzelne Währungen wie Euro oder US-Dollar umgetauscht werden können. So sind diese Libra-Stablecoins aus Sicht der Nutzer als eine spezielle Form von Giralgeld und die Libra Association letztlich nur als ein Zahlungsdienstleister wie etwa PayPal anzusehen. Der komparative Vorteil von Libra könnte dann insbesondere in seiner Programmierbarkeit und in dem damit entstehenden Finanz-Ökosystem liegen. Letzteres wird besonders im Bereich der grenzüberschreitenden Zahlungen deutlich. Eine Überweisung von Deutschland nach Mexiko wird zum aktuellen Zeitpunkt immer mit einem Umtausch von Euro in Mexikanischen Peso einhergehen. Mit Libra wäre es allerdings denkbar, einen Libra-Euro einem Libra-Konto in Mexiko gutzuschreiben. Dieser Libra-Euro könnte dann bei vielen Anbietern (dem Ökosystem) unabhängig von der nationalen Währung als Zahlungsmittel genutzt werden.

Faktisch könnte der Euro somit auch außerhalb des Euroraums als Zahlungsmittel genutzt werden. Libra wäre in diesem Falle ein sehr enges Substitut zum bestehenden Euro-Giralgeld. Wie bei Geschäftsbanken können Libra-Guthaben transferiert, als Zahlungsmittel genutzt, oder zu Bargeld umgetauscht werden. Folglich stehen vor allem Geschäftsbanken unter Zugzwang, durch Produktinnovation die eigene Effizienz im Zahlungsverkehr zu erhöhen, um weiter konkurrenzfähig zu bleiben. Entsprechend sieht Jens Weidmann, Präsident der Deutschen Bundesbank, Libra vor allem als Konkurrenz für Zahlungsdienstleistungen der Geschäftsbanken und fordert Banken auf, durch eigene Innovationen im Zahlungsverkehr digitale Alternativen zu schaffen (PYMNTS.com, 2020). In einer Marktwirtschaft seien laut Weidmann in erster Linie private Unternehmen, nicht der öffentliche Sektor, gefordert, um sich veränderter Nachfrage durch neue Angebote anzupassen.

Aus der Perspektive der Regulatoren und Zentralbanker in Industrieländern ergeben sich daraus drei grundsätzliche Konsequenzen:

1. Die Libra Association ist „nur“ ein konventioneller Zahlungsdienstleister mit neuer Organisationsform und nutzerfreundlicher Integration in soziale Netzwerke und damit entsprechend wie andere Zahlungsdienstleister zu regulieren. Dies bedeutet unter anderem, dass Nutzer klassische Know-your-customer-Prozesse durchlaufen müssten, um ein Konto eröffnen zu können.

2. Als enges Giralgeld-Substitut wäre Libra der Geldmenge M3 zuzurechnen. Für die monetäre Analyse von Zentralbanken ist eine statistische Berücksichtigung von Libra-Konten daher unerlässlich. Sollten Libras Integration in soziale Netzwerke und ihre Programmierbarkeit tatsächlich wie geplant vonstatten gehen und Transaktionen für Nutzer erheblich bequemer ausgestalten sein, ist nicht auszuschließen, dass Libra eine sehr hohe Marktkapitalisierung erreichen und damit einen erheblichen Teil der Geldmenge M3 ausmachen könnte. In einer aktuellen Studie diskutiert die EZB unterschiedliche Szenarien mit einem Marktpotenzial für Libra von 150 Mrd. Euro bis zu 3.000 Mrd. Euro (EZB, 2020a).

3. Die Wertstabilität soll dadurch abgesichert werden, dass für neu geschaffene Libra eine sogenannte Libra-Reserve aus Bankdepositen und hochliquiden Staatsschuldentiteln hinterlegt wird. Bankdepositen spielen daher für Libra eine ähnliche Reserverolle wie Zentralbankgeld für private Bankdepositen. Damit wird das bisherige zweistufige Geldsystem aus Zentralbankgeld und Bankdepositen um die von privaten Nichtbanken geschaffene Libra zu einem dreistufigen System erweitert. Entwicklungen im Libra-Bereich wären aufgrund dieser Verbindungen auch unmittelbar für Geschäftsbanken, Notenbanken und Bankregulierung relevant.

Neben diesem Aspekt der Finanzmarktstabilität könnte für Zentralbanken angesichts neuer technologischer Möglichkeiten und erheblicher Nachfrage nach digitalen Zahlungsmitteln auch die Frage relevant werden, ob sie digitales Zentralbankgeld als Pendant zu physischem Zentralbankgeld, d.h. Banknoten und Münzen, anbieten sollten. Dies hätte einige Vorteile: Mit CBDC könnten Notenbanken die Geldversorgung digital sicherstellen und die hohen Kosten für Produktion, Erhaltung, Verteilung und Überwachung von Bargeld vermeiden. Zudem müssen Zentralbanken laut Mandat gewährleisten, dass der Zahlungsverkehr als öffentliche Aufgabe auch bei schwindender Bargeldverwendung sichergestellt und nicht gänzlich abhängig von privaten Anbietern ist. Weiterhin sind digitale Transaktionen leichter zu überwachen und bieten ein Mittel im Kampf gegen Geldwäsche und Terrorismusfinanzierung. Dies gilt allerdings nur, wenn CBDC-Transaktionen nachverfolgt werden können und Zugang zu der CBDC nur nach Know-your-customer-Prozessen möglich ist. CBDC ließe sich außerdem – anders als Bargeld – verzinsen. Zinsen können CBDC als Zahlungsmittel bzw. als Wertaufbewahrungsmittel mehr oder weniger attraktiv machen und eröffnen somit neue Möglichkeiten für die Geldpolitik. Dazu gehören nicht zuletzt auch Negativzinsen. Zudem würde der CBDC-Zins im Falle einer zinstragenden CBDC für alle Halter von Zentralbankgeld simultan gelten und nicht wie bisher nur für die Einlagen der Banken bei der Notenbank. Dies könnte die Effizienz der geldpolitischen Transmission erhöhen.

Die großen Vorteile von digitalem Zentralbankgeld haben Zentralbanken dazu bewogen, sich intensiv mit dem Thema auseinanderzusetzen. Während einige Zentralbanken, wie beispielsweise die EZB, zunächst noch Möglichkeiten und Risiken von CBDC ausloten, arbeiten andere bereits konkret an der Umsetzung oder sind bereits in der Testphase. So planen weltweit rund ein Fünftel der Zentralbanken innerhalb der nächsten sechs Jahre eine CBDC für die breite Öffentlichkeit (Retail CBDC) einzuführen (Boar, Holden und Wadsworth, 2020). Dazu gehören beispielsweise die Marshall Islands, die schon innerhalb der nächsten zwei Jahre eine eigene digitale Währung emittieren wollen, und die chinesische Zentralbank, die bereits heute im Rahmen einer Testphase Mitarbeitern im öffentlichen Dienst regional beschränkt Teile des Gehalts in CBDC auszahlt.

## Geldsystem der Zukunft

Im aktuellen Innovationsprozess stellen sich einige Fragen: Welche Rolle werden neue Zahlungsmittel wie Bitcoin, Libra und CBDC im zukünftigen Geldsystem einnehmen? Wie werden die (Konkurrenz-)Beziehungen zwischen den neuen Geldformen ausgestaltet? Welche Folgen ergeben sich für die Politik der Zentralbanken?

Die Rolle von nicht-besicherten Kryptowährungen ist relativ gut abschätzbar, nicht zuletzt aufgrund der schon vergleichsweise großen Erfahrungen mit Bitcoin. Diese Währung wird gänzlich außerhalb des konventionellen Finanzsystems produziert und ist derzeit aufgrund der hohen Volatilität nicht als direkter Konkurrent für alltägliche Zahlungsmittel wie Bargeld und Bankeinlagen anzusehen. Eher wird Bitcoin als digitaler Wertspeicher und Spekulationsobjekt genutzt. Daneben ist ein kaum überschaubares Spektrum an Kryptowährungen entstanden. Quantitativ ist ihr Einfluss aber immer noch relativ klein. Dennoch hat sich mit Bitcoin trotz Problemen ein paralleles privates Zahlungssystem entwickelt, das in den letzten Jahren immer mehr Zuspruch erfahren hat. Seine Bedeutung dürfte vor allem darin liegen, dass ein Weg im Sinne eines „Proof of Principle" aufgezeigt wurde, privates Geld unabhängig vom konventionellen zweistufigen Bankensystem zu schaffen. Möglicherweise könnten Kryptowährungen auch im Falle systemischer Probleme von Fiat-Währungen eine besondere Relevanz gewinnen.

Dagegen handelt es sich bei Libra – trotz allen intensiven technologie-affinen Marketings – um ein vergleichsweise konventionelles Finanzprodukt, das eng mit dem bestehenden Finanzsystem verbunden ist. Insbesondere sollen Bankdepositen eine Reserverolle für Libra spielen, ähnlich den Zentralbankreserven für die Bankdepositen. Das derzeitige zweistufige Geldsystem würde durch Libra zu einem dreistufigen Geldsystem erweitert. Diese Nähe von Libra zu konventionellen Finanzprodukten wirft damit auch vielfältige geldpolitische und regulatorische Fragen auf. Insbesondere ist es unabdingbar, dass Libra wie vergleichbare Bankprodukte behandelt und reguliert wird.

Von der Libra Association wurde zudem die Möglichkeit ins Spiel gebracht, dass von der Notenbank angebotene CBDC, etwa eine von der Europäischen Zentralbank angebotene Euro-CBDC, zukünftig in das Libra-System integriert werden können. Die Libra Association würde dann entsprechend Single-Currency-Libra in der gleichen Währung anbieten, die ausschließlich durch entsprechende CBDC gedeckt wären und nicht mehr durch Bankdepositen und liquide Staatsschuldentitel. Eine solche Libra auf Basis einer CBDC wäre ein sehr enges Substitut nicht nur zu Bankdepositen, sondern auch zu Bargeld. Da vollständig durch Zentralbankgeld hinterlegt, wäre das Libra-System faktisch ein Vollgeldsystem. Allerdings ist derzeit nicht absehbar, ob Zentralbanker diese Ausgestaltung in der Praxis unterstützen würden.

Sollten Notenbanken CBDC als digitales Bargeld anbieten, ist eine breite Nutzung als Zahlungsmittel zu erwarten. Insbesondere würden CBDC wohl auch als gesetzliches Zahlungsmittel gelten. Allerdings ist zum aktuellen Zeitpunkt nicht absehbar, wann es zu einem solchen Angebot kommen wird. Im Fall der Europäischen Zentralbank scheint eine CBDC-Einführung laut EZB-Direktoriumsmitglied Yves Mersch aufgrund der weiterhin wichtigen Stellung von Bargeld in naher Zukunft unwahrscheinlich (EZB, 2020b). Allerdings ist nicht auszuschließen, dass Bargeld, auch getrieben durch die immer größere Verbreitung von digitalen Bezahlmethoden infolge der Covid-19-Pandemie, in den nächsten Jahren an Bedeutung verlieren und eine CBDC-Einführung entsprechend wahrscheinlicher werden könnte.
